# Clinical predictive factors and prediction models for end‐stage renal disease in Chinese patients with type 2 diabetes mellitus

**DOI:** 10.1002/ctm2.1323

**Published:** 2023-06-29

**Authors:** Yueming Gao, Zhi Shang, Sheng Nie, Songtao Feng, Bin Wang, Zuolin Li, Min Wu, Yi Wen, Hong Xu, Jianping Weng, Chunbo Chen, Huafeng Liu, Qiongqiong Yang, Hua Li, Yaozhong Kong, Guisen Li, Qijun Wan, Yan Zha, Ying Hu, Gang Xu, Yongjun Shi, Yilun Zhou, Guobin Su, Ying Tang, Mengchun Gong, Hou Fan Fan, Bicheng Liu

**Affiliations:** ^1^ Institute of Nephrology Zhongda Hospital, Southeast University School of Medicine Nanjing China; ^2^ Department of Nephrology Peking University Third Hospital Beijing China; ^3^ Department of Cardiology and Institute of Vascular Medicine Peking University Third Hospital Beijing China; ^4^ Division of Nephrology, Nanfang Hospital Southern Medical University; National Clinical Research Center for Kidney Disease; State Key Laboratory of Organ Failure Research; Guangdong Provincial Institute of Nephrology; Guangdong Provincial Key Laboratory of Renal Failure Research Guangzhou China; ^5^ Children's Hospital of Fudan University Shanghai China; ^6^ Department of Endocrinology The First Affiliated Hospital of USTC, Division of Life Sciences and Medicine, University of Science and Technology of China Hefei China; ^7^ Department of Critical Care Medicine Maoming People's Hospital Maoming China; ^8^ Key Laboratory of Prevention and Management of Chronic Kidney Disease of Zhanjiang City Institute of Nephrology, Affiliated Hospital of Guangdong Medical University Zhanjiang China; ^9^ Department of Nephrology Sun Yat‐Sen Memorial Hospital, Sun Yat‐Sen University Guangzhou China; ^10^ Sir Run Run Shaw Hospital Zhejiang University School of Medicine Hangzhou China; ^11^ Department of Nephrology The First People's Hospital of Foshan Foshan China; ^12^ Renal Department and Institute of Nephrology Sichuan Provincial People's Hospital, School of Medicine, University of Electronic Science and Technology of China, Sichuan Clinical Research Center for Kidney Diseases Chengdu China; ^13^ The Second People's Hospital of Shenzhen, Shenzhen University Shenzhen China; ^14^ Guizhou Provincial People's Hospital, Guizhou University Guiyang China; ^15^ The Second Affiliated Hospital of Zhejiang University School of Medicine Hangzhou China; ^16^ Division of Nephrology Tongji Hospital, Tongji Medical College, Huazhong University of Science and Technology Wuhan China; ^17^ Huizhou Municipal Central Hospital Sun Yat‐Sen University Huizhou China; ^18^ Department of Nephrology Beijing Tiantan Hospital, Capital Medical University Beijing China; ^19^ Department of Nephrology Guangdong Provincial Hospital of Chinese Medicine, The Second Affiliated Hospital, The Second Clinical College, Guangzhou University of Chinese Medicine Guangzhou China; ^20^ The Third Affiliated Hospital of Southern Medical University Guangzhou China; ^21^ Institute of Health Management Southern Medical University Guangzhou China; ^22^ Digital Health China Technologies Co., LTD Beijing China

Dear Editor

Diabetes mellitus (DM) has become a significant chronic condition that seriously affects human health.^1^ Nowadays, China has become the country with the largest number of DM patients worldwide, of which more than 90% are type 2 diabetes mellitus (T2DM).^2^ The increasing prevalence of DM exacerbates the incidence of end‐stage renal disease (ESRD).^3^ T2DM‐related ESRD not only reduces survival rate and health‐related quality of life but also places a significant cost on patients as well as society.^4^, ^5^, ^6^

To identify clinical predictive factors and develop prediction models for ESRD risk in T2DM patients, we used the study population extracted from the China Renal Data System, a database containing the information of more than seven million patients attended at 19 hospitals in the Chinese mainland, as previously described.^7^ ESRD, including an eGFR of 15 mL/min/1.73 m^2^ or less, or the commencement of dialysis or kidney transplantation due to ESRD, was classified as the outcome. Eventually, clinical data of adult patients with T2DM were collected from 17 hospitals. Using a randomized approach, 55 824 patients with T2DM from 10 medical centers were included in the derivation cohort, and 25 745 patients from seven additional medical institutions were included for external validation. The patient selection flowchart is shown in Figure S1.

After a median of 384 (123, 900) days of follow‐up, there were 1,527 (2.74%) outcomes in the derivation cohort (*n* = 55,824). Table S1 summarizes the clinical features at baseline. Spearman correlation analysis was conducted to identify the correlation between continuous variables (Figure S2), and variables with higher average correlation (correlation ≥ 0.5) were removed. Univariate Cox regression analysis was used to select potential predictors (*p* < 0.1), as demonstrated in Table S2. All potential predictors were, therefore, fitted into a multivariable Cox regression model, utilizing step‐wise backward selection (*p* < 0.05). Ten clinical predictive factors, including age, hypertension, diabetes retinopathy (DR), hemoglobin (HGB), serum albumin (ALB), serum creatinine (Scr), serum uric acid, Low‐density lipoprotein cholesterol (LDL‐C), serum fibrinogen, and urinary protein were selected into the final model (Table S3). We constructed three clinical prediction models using various combinations of predictors selected by multivariable Cox regression (Table 1). Model 1 (full model), including all of the 10 clinical predictive factors, achieved the highest discrimination (area under the curve [AUC]: 0.926, 95% confidence interval [CI]: 0.919–0.934); model 2 (laboratory model), including all the laboratory indicators, with an AUC of 0.924 (95% CI: 0.917–0.932); model 3 (simplified model) incorporated five easily accessible predictors, including age, hypertension, HGB, Scr, and urinary protein, with an AUC of 0.916 (95% CI: 0.908–0.924) (Figure 1A). In addition, these models attained satisfactory calibration, as shown in Figure S3A‐C. Internal validation using bootstrapping also achieved a robust discrimination, with an AUC of 0.914–0.927, as shown in Table 1.

**TABLE 1:** Clinical prediction models with different combinations of predictors.

Predictors	Model 1 (full model)	Model 2 (laboratory model)	Model 3 (Simplified model)
Age (incremented by 1 year)	0.993	/	.988
Hypertension	1.651	/	1.829
DR	1.433	/	/
HGB (incremented by 1 g/L)	0.982	0.982	.980
Serum ALB (incremented by 1 g/L)	0.962	0.963	/
Scr (incremented by 1 μmol/L)	1.009	1.009	1.010
Serum uric acid (incremented by 1 μmol/L)	1.001	1.001	/
LDL‐C (incremented by 1 mmol/L)	1.091	1.110	/
Serum fibrinogen (incremented by 1 g/L)	1.055	1.070	/
Urinary protein^†^	4.608	5.159	6.000
Urinary protein^‡^	7.647	8.930	12.309
		/	/
AUC (derivation cohort)	0.926 (0.919, 0.934)	0.924 (0.917, 0.932)	.916 (0.908, 0.924)
AUC (internal derivation cohort)	0.927 (0.920, 0.935)	0.925 (0.917, 0.932)	0.914 (0.906, 0.922)
AUC (eternal validation cohort)	0.882 (0.871, 0.894)	0.877 (0.866, 0.889)	0.868 (0.856, 0.881)

Abbreviations: ALB, albumin; AUC, area under the curve; DR, diabetic retinopathy; HGB, hemoglobin; LDL‐C, low‐density lipoprotein cholesterol; Scr, serum creatinine.

^†^ The level of urine protein was 1+ or 2+.

^‡^ The level of urine protein was 3+ or 4+.

**FIGURE 1:**
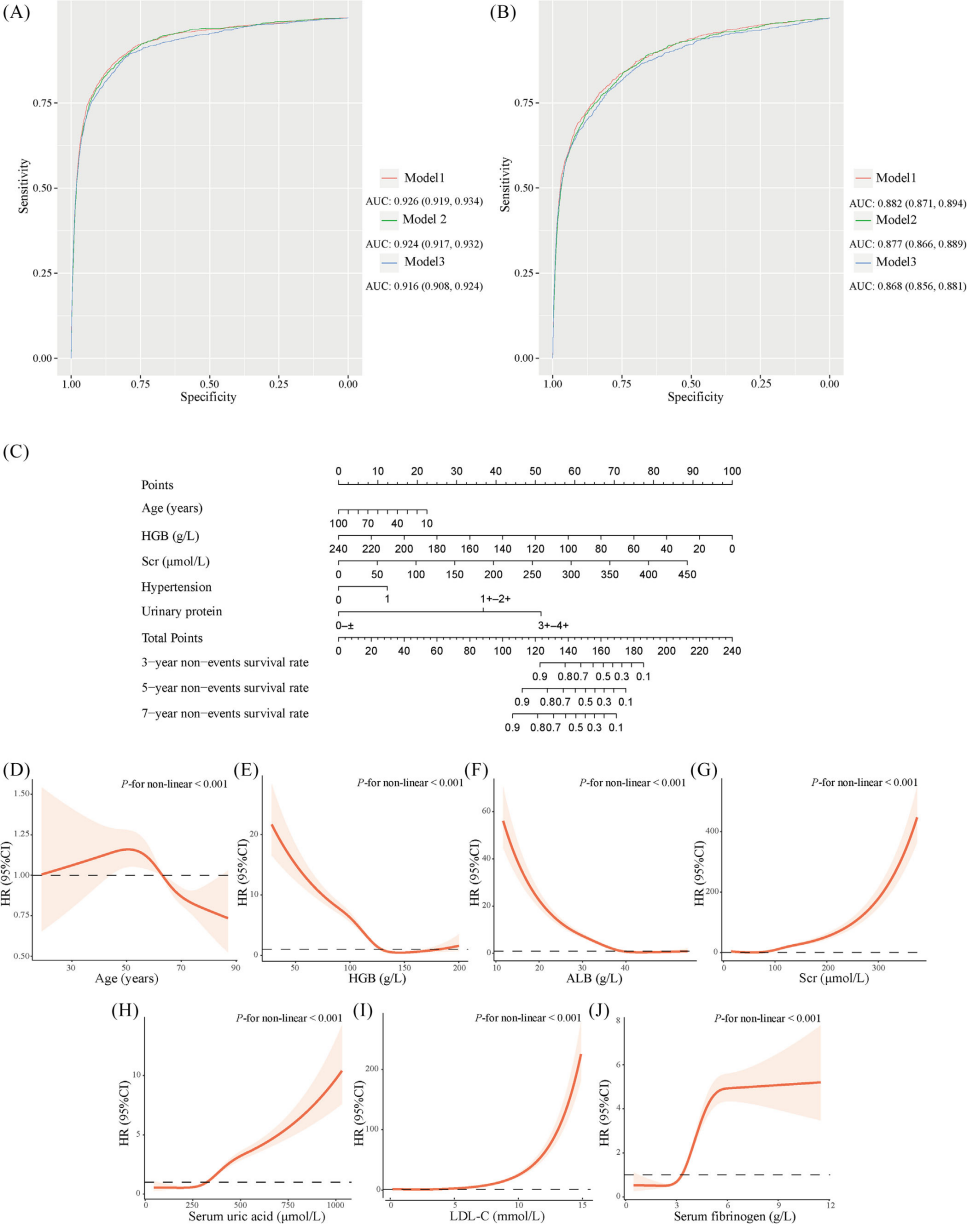
(A) Receiver operating characteristic (ROC) curves for different clinical prediction models in the derivation cohort. (B) ROC curves for different clinical prediction models in the validation cohort. (C) A nomogram of model 3 (simplified model). (D–J) Restricted cubic spline (RCS) curves of different continuous variables in model 1(full model).

In the external validation cohort (*n* = 25,745), during a median follow‐up of 321 (90, 758) days, there were 1,084 (4.21%) outcomes. Table S4 presents the baseline clinical features. Based on the receiver‐operating characteristic (ROC) curves, the prediction models achieved an AUC ranging from 0.868 to 0.882 (Figure 1B). As seen in Figure S3D‐F, these models also attained satisfactory calibration.

Also, we illustrated model 3 (simplified model) as a nomogram (Figure 1C). Additionally, we determined the appropriate cut‐off values for the seven continuous variables included in model 1 (full model) using a minimum *p*‐value method (Table S5). We then employed restricted cubic spline (RCS) to describe the relationship between these clinical predictors and outcomes, as illustrated in Figure 1D–J. Based on the findings of RCS curves and the minimum *p*‐value method, as well as the clinical application practicality, the seven continuous variables in model 1 were categorized into different categories. Then, multivariate cox regression was conducted, and a score was awarded to each variable based on the hazard ratio (HR) presented in Table S6. Finally, a risk score was developed as follows: age (year; ≥ 56 scores 0 and < 56 scores 1), hypertension (yes scores 2 and no scores 0), DR (yes scores 1 and no scores 0), HGB (g/L; ≥ 108 scores 0 and < 108 scores 2), serum ALB (g/L; ≥ 33 scores 0 and < 33 scores 2), Scr (μmol/L; < 115 scores 0 and ≥ 115 scores 5), serum uric acid (μmol/L; < 435 scores 0 and ≥ 435 scores 1), LDL‐C (mmol/L; < 4 scores 0 and ≥ 4 scores 1), serum fibrinogen (g/L; < 4 scores 0 and ≥ 4 scores 1) and urinary protein (0 – ± scores 0, 1+−2+ scores 4 and 3+−4+ scores 8), as shown in Table 2.

**TABLE 2:** A risk score of model 1(full model).

Predictors	Category	Point
Age (year)	≥56	0
	<56	1
Hypertension	No	0
	Yes	2
DR	No	0
	Yes	1
HGB (g/L)	≥108	0
	<108	2
Serum ALB (g/L)	≥33	0
	<33	2
Scr (μmol/L)	<115	0
	≥115	5
Serum uric acid (μmol/L)	<435	0
	≥435	1
LDL‐C (mmol/L)	<3.4	0
	≥3.4	1
Serum fibrinogen (g/L)	<4	0
	≥4	1
Urinary protein	0 – ±	0
	1+ – 2+	4
	3+ – 4+	8

Abbreviations: ALB, albumin; DR, diabetic retinopathy; HGB, hemoglobin; LDL‐C, low‐density lipoprotein cholesterol; Scr, serum creatinine.

Furthermore, we categorized the patients with T2DM into four risk groups according to the RCS curve (Figure 2A), including the low‐risk (total points < 8), moderate‐risk (8 ≤ total points < 15), high‐risk (15 ≤ total points < 20) and very high‐risk group (20 ≤ total points < 24), as shown in Figure 2B. As depicted by Kaplan–Meier survival curves (Figure 2C,D), in the derivation cohort, the HRs of developing ESRD in the moderate‐, high‐, and very high‐risk groups were 18.64 (95% CI: 15.97–21.76), 80.62 (95% CI: 69.91‐‐94.33) and 178.16 (95% CI: 148.66‐‐213.52) in comparison with the low‐risk group (*p* < 0.001); for patients in the validation cohort, the HRs in the moderate‐, high‐, and very high‐risk groups were 10.95 (95% CI: 9.347–12.82), 44.18 (95% CI: 37.51–52.05) and 89.59 (95% CI: 72.47–110.74) in comparison with the low‐risk group (*p* < 0.001).

**FIGURE 2:**
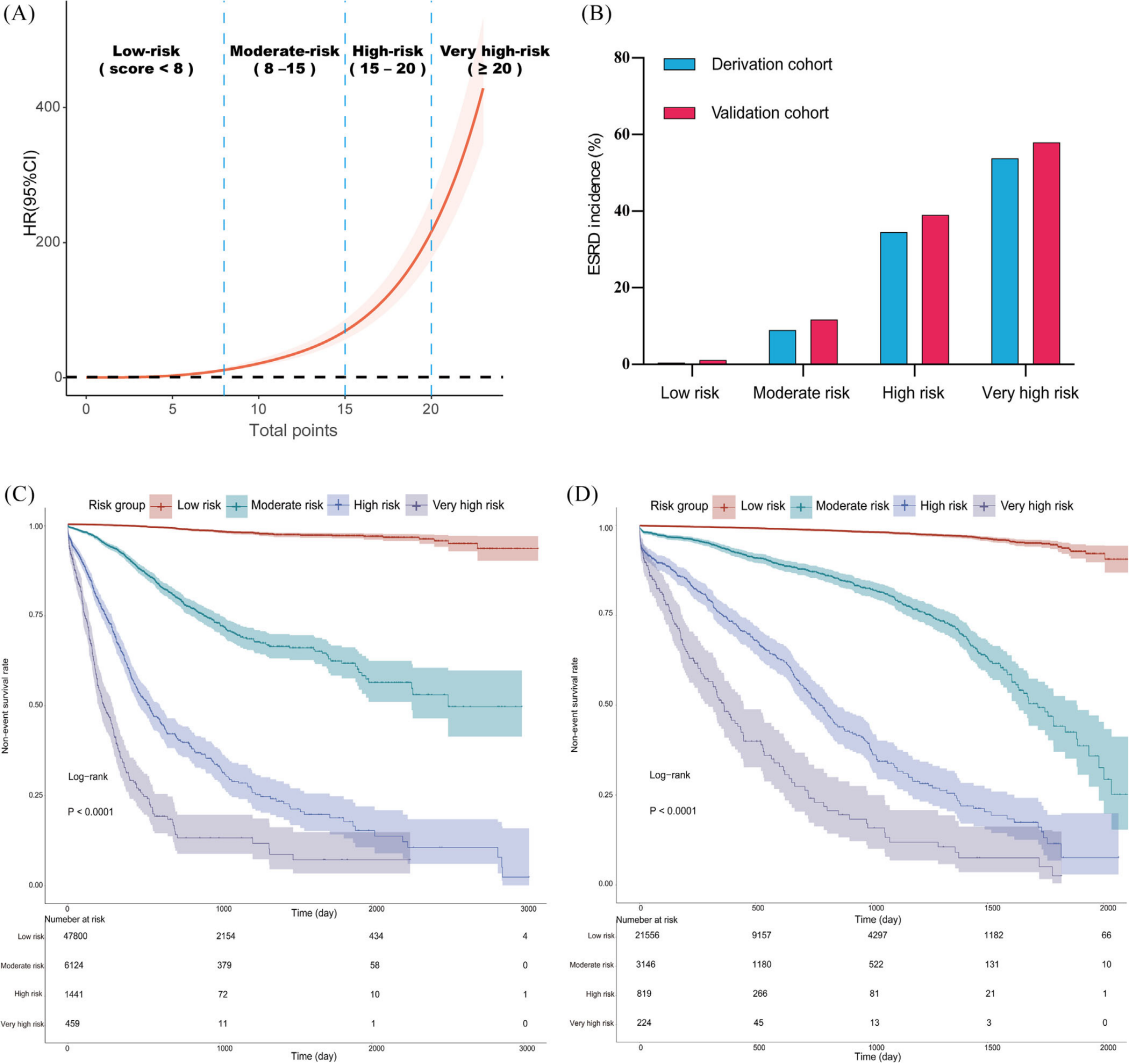
(A) Restricted cubic spline (RCS) curves of the total points of the risk score model. (B) Risk stratification based on the risk score model in the derivation and the validation cohort. (C–D) Kaplan–Meier curve of each risk group in the derivation and the validation cohort.

In conclusion, using a large multi‐center retrospective cohort in the Chinese mainland, we identified 10 clinical predictive factors and developed models to predict ESRD in T2DM patients, which showed excellent prediction performance. To the best of our knowledge, we have established models to predict ESRD based on the largest population of T2DM patients in the Chinese Mainland. These prediction models were further provided as simple bedside tools, including a risk score and a nomogram, which could be extensively applied to assess T2DM patients’ ESRD risk in clinical practice, to aid clinical decision‐making and sensible resource allocation.

## CONFLICT OF INTEREST STATEMENT

The authors declare no conflict of interest.

## Supporting information

Supporting Information

## Data Availability

The data that support the findings of this study are accessible from the corresponding author upon request. The data are not publicly available owing to ethical or privacy concerns.
