# Supplementary material for: Clinical predictive factors and prediction models for end‐stage renal disease in Chinese patients with type 2 diabetes mellitus
Source: Clin Transl Med. 2023 Jun 29;13(7):e1323. doi: 10.1002/ctm2.1323 (PMC10310973; doi:10.1002/ctm2.1323)
Supplement: Supplementary file 1 — Supporting Information [file CTM2-13-e1323-s001.docx]

**Supplemental Materials**

**
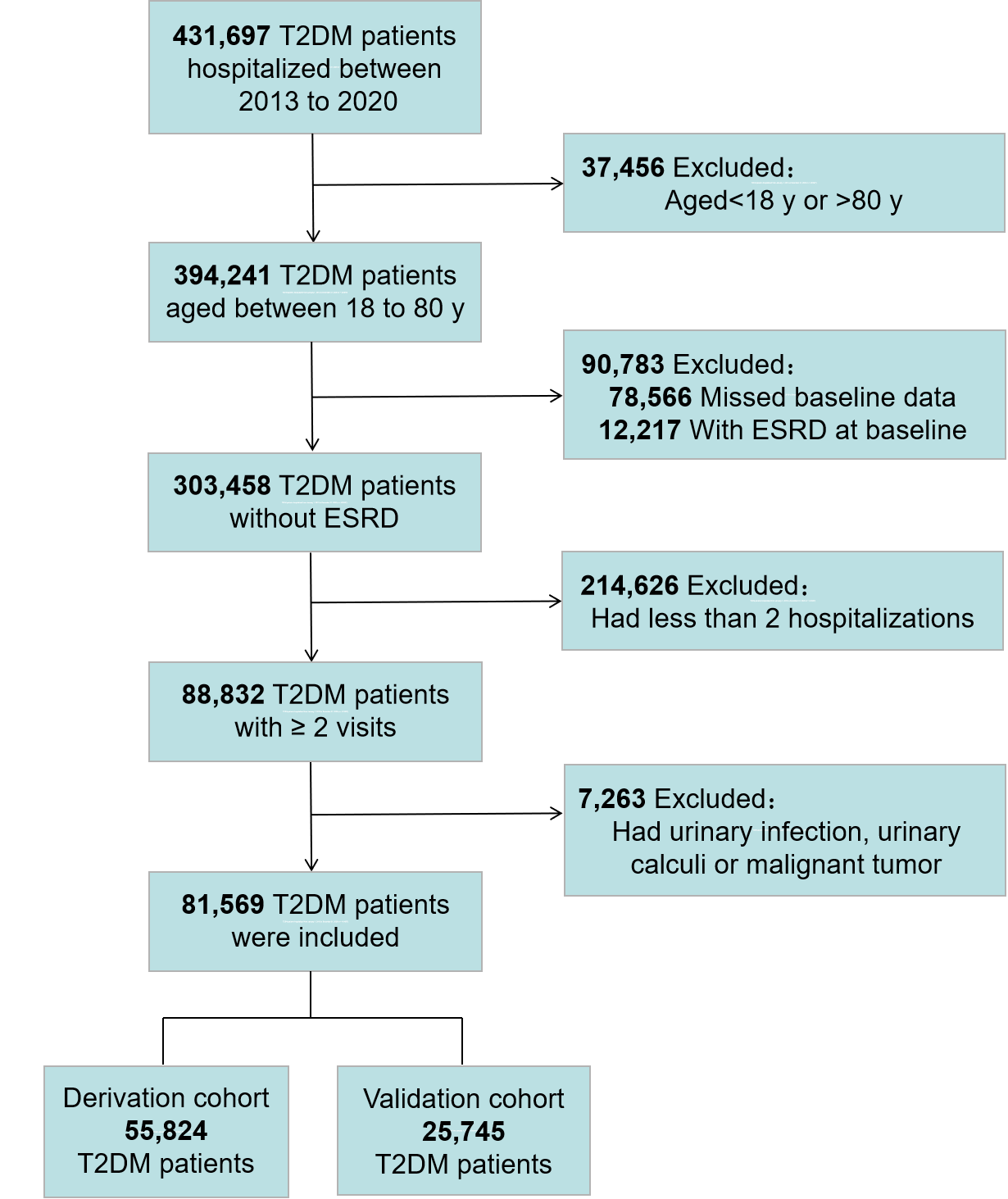
**

**Figure S1.** Flowchart of patient selection. Abbreviations: T2DM: type 2 diabetes mellitus; ESRD: end-stage renal disease.


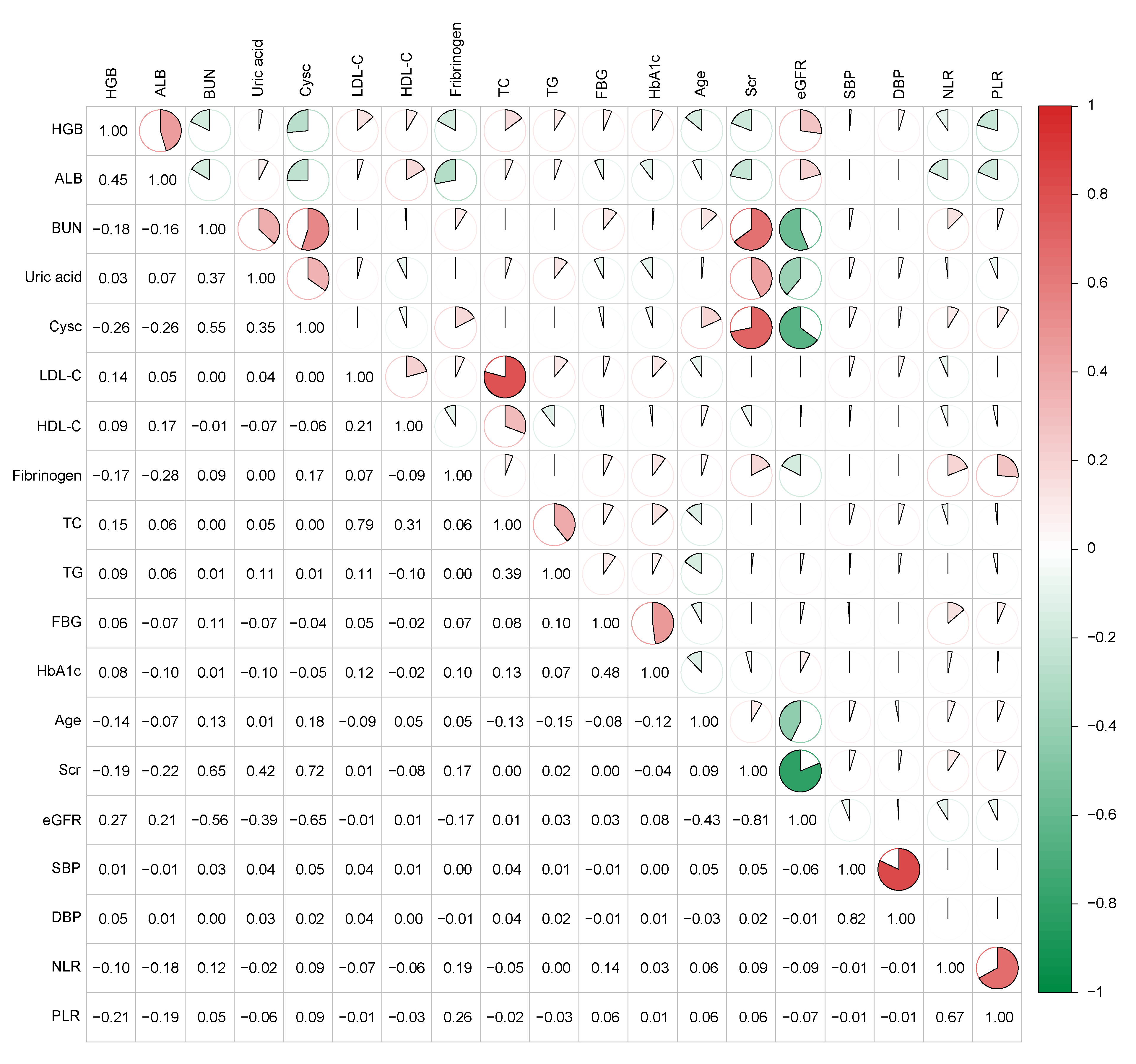


**Figure S2.** Spearman correlation analysis between continuous variables in the derivation cohort. Consequently, eGFR, serum CysC, TC, NLR, and DBP were taken out of subsequent analysis due to the r-value > 0.5.


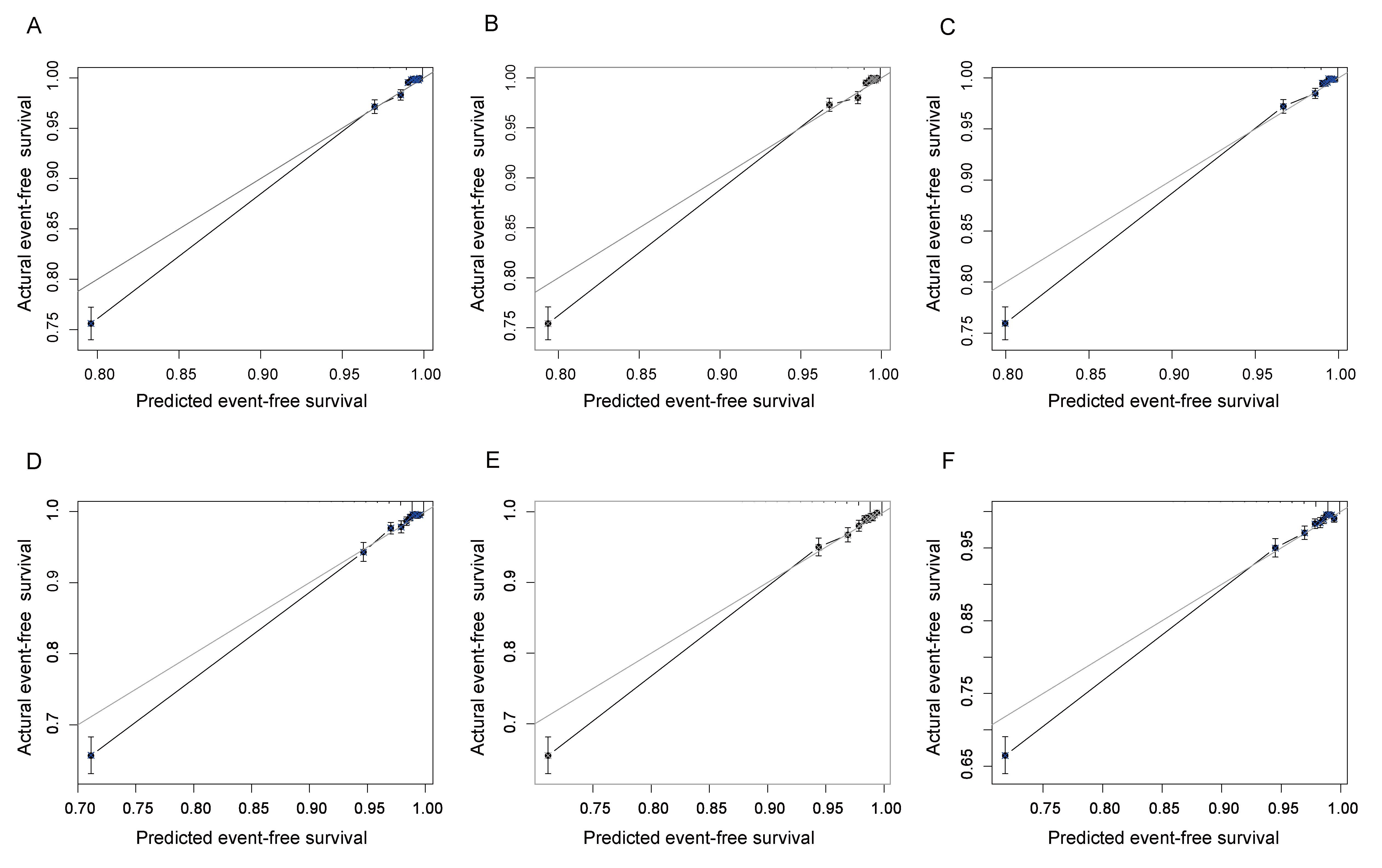


**Figure S3.** The calibration curves for different clinical prediction models in the derivation and the validation cohort. (A)-(C): calibration curves for model 1 (full model), model 2 (laboratory model), and model 3 (simplified model) in the derivation cohort; (D)-(F): calibration curves for model 1, model 2 and model 3 in the derivation cohort. Bootstrapping method was used to get bias-corrected estimates of predicted *vs.* observed values based on subsetting predictions into intervals. The calibration plot showed the agreement between the predicted probability (x-axis) and the actual probability (y-axis) of the 24-month event-free survival rate. A perfect prediction would correspond to the grey line.

**Table S1.** Baseline clinical characteristics of patients in the derivation cohort

| **Characteristics** | **Overall**  **(n= 55,824)** | **Patients**  **without outcome**  **(n = 54,297)** | **Patients**  **with outcome**  **(n = 1,527)** | ***P*-value** |
| --- | --- | --- | --- | --- |
| **Demographics** | | | | |
| Age (years) | 62 ± 11 | 62 ± 11 | 61 ± 11 | <0.001 |
| Sex (male %) | 58.40 | 58.22 | 64.70 | <0.001 |
| **Complications or Comorbidities** | | | | |
| DR (%) | 8.16 | 7.47 | 32.81 | <0.001 |
| Diabetic peripheral neuropathy (%) | 11.20 | 10.81 | 25.34 | <0.001 |
| Diabetic vascular disease (%) | 5.57 | 5.37 | 12.51 | <0.001 |
| Hypertension (%) | 55.91 | 55.23 | 80.29 | <0.001 |
| CHD (%) | 22.08 | 22.09 | 21.81 | 0.817 |
| Cerebral vascular disease (%) | 19.48 | 19.45 | 20.83 | 0.191 |
| **Treatment method** | | | | |
| Insulin (%) | 46.39 | 45.86 | 65.09 | <0.001 |
| DPP-4 inhibitors (%) | 3.36 | 3.38 | 2.55 | 0.091 |
| GLP-1RA (%) | 0.52 | 0.52 | 0.26 | 0.221 |
| SGLT-2 inhibitors (%) | 0.23 | 0.23 | 0.00 | 0.107 |
| RAAS inhibitors (%) | 19.05 | 18.53 | 37.59 | <0.001 |
| Lipid-lowering agents (%) | 22.55 | 22.32 | 30.78 | <0.001 |
| **Physical and laboratory findings** | | | | |
| SBP (mmHg) | 135 (121, 149) | 134 (121, 148) | 149 (133, 167) | <0.001 |
| DBP (mmHg) | 80 (71, 88) | 80 (71, 87) | 83 (74, 92) | <0.001 |
| HGB (g/L) | 126.72 ± 21.91 | 127.28 ± 21.62 | 106.77 ± 22.72 | <0.001 |
| Neutrophils (×10^9^/L) | 4.14 (3.11, 5.67) | 4.12 (3.10, 5.64) | 4.82 (3.64, 6.39) | <0.001 |
| Lymphocytes (×10^9^/L) | 1.72 (1.27, 2.22) | 1.72 (1.27, 2.22) | 1.59 (1.19, 2.07) | <0.001 |
| Platelets (×10^9^/L) | 216.50 ± 87.52 | 216.05 ± 87.44 | 232.73 ± 89.00 | <0.001 |
| NLR | 2.36 (1.64, 3.70) | 2.34 (1.63, 3.67) | 2.95 (2.07, 4.41) |  |
| PLR | 118.81  (87.93, 165.39) | 118.39  (87.57, 164.56) | 139.61  (102.05, 188.69) | <0.001 |
| Serum fibrinogen (g/L) | 3.58 ± 1.28 | 3.56 ± 1.27 | 4.46 ± 1.40 | <0.001 |
| Serum ALB (g/L) | 38.41 ± 5.40 | 38.58 ± 5.26 | 32.33 ± 6.59 | <0.001 |
| Scr (μmol/L) | 77.73 ± 39.00 | 75.07 ± 33.60 | 172.32 ± 79.16 | <0.001 |
| BUN (mmol/L) | 5.97 ± 2.84 | 5.84 ± 2.65 | 10.57 ± 4.89 | <0.001 |
| Serum uric acid (μmol/L) | 336.32 ± 116.87 | 333.74 ± 115.49 | 428.01 ± 128.25 | <0.001 |
| Serum CysC (mg/L) | 1.11 ± 0.50 | 1.08 ± 0.45 | 2.14 ± 0.95 | <0.001 |
| eGFR (mL/min/1.73 m^2^) | 87.34±26.58 | 89.03±25.01 | 49.09±32.09 | <0.001 |
| LDL-C (mmol/L) | 2.73 ± 1.06 | 2.72 ± 1.04 | 3.16 ± 1.44 | <0.001 |
| HDL-C (mmol/L) | 1.05 ± 0.32 | 1.05 ± 0.32 | 1.06 ± 0.36 | 0.053 |
| TC (mmol/L) | 4.55 ± 1.42 | 4.54 ± 1.40 | 5.21 ± 1.95 | <0.001 |
| TG (mmol/L) | 1.45 (1.03, 2.12) | 1.44 (1.03, 2.11) | 1.66 (1.16, 2.42) | <0.001 |
| FBG (mmol/L) | 8.89 ± 4.37 | 8.89 ± 4.34 | 8.81 ± 5.31 | 0.572 |
| HbA1c (%) | 7.98 ± 2.03 | 7.97 ± 2.03 | 8.03 ± 2.31 | 0.336 |
| Urinary protein |  |  |  |  |
| 0~± (%) | 84.87 | 86.61 | 22.98 | <0.001 |
| 1+ – 2+ (%) | 12.20 | 11.30 | 44.34 | <0.001 |
| 3+ – 4+ (%) | 2.93 | 2.09 | 32.68 | <0.001 |

Abbreviations: DR: diabetic retinopathy; CHD: coronary heart disease; DPP-4: dipeptidyl peptidase-4; GLP1-RA: glucagon-like peptide receptor agonists; SGLT-2: sodium-glucose co-transporter 2; RAAS: renin-angiotensin-aldosterone system; SBP: systolic blood pressure; DBP: diastolic blood pressure; HGB: hemoglobin; NLR: neutrophil/lymphocyte ratio; PLR: platelet/lymphocyte ratio; ALB: albumin; Scr: serum creatinine; BUN: blood urea nitrogen; CysC: cystatin C; eGFR: estimated glomerular filtration rate; LDL-C: low-density lipoprotein cholesterol; HDL-C: high-density lipoprotein cholesterol; TC: total cholesterol; TG: triglyceride; FBG: fasting blood glucose; HbA1c: glycated hemoglobin.

**Table S2**. Potential predictors identified by univariate Cox regression analysis

| **Predictors** | **HR** | **95%CI** | ***P*-value** |
| --- | --- | --- | --- |
| Age (incremented by 1 year) | 0.989 | 0.985-0.994 | <0.001 |
| Sex (male %) | 1.352 | 1.217-1.501 | <0.001 |
| Hypertension | 3.072 | 2.708-3.485 | <0.001 |
| DR | 4.239 | 3.808-4.719 | <0.001 |
| Diabetic peripheral neuropathy | 1.819 | 1.620-2.043 | <0.001 |
| Diabetic vascular disease | 1.938 | 1.665-2.256 | <0.001 |
| Insulin | 1.935 | 1.741-2.149 | <0.001 |
| GLP1-RA | 0.383 | 0.144-1.022 | 0.055 |
| RAAS ihibitors | 2.143 | 1.932-2.378 | <0.001 |
| Lipid-lowering agents | 1.383 | 1.240-1.542 | <0.001 |
| HGB (incremented by 1 g/L) | 0.963 | 0.962-0.965 | <0.001 |
| PLR (incremented by 1) | 1.002 | 1.002-1.002 | <0.001 |
| Serum ALB (incremented by 1 g/L) | 0.853 | 0.848-0.859 | <0.001 |
| Scr (incremented by 1 μmol/L) | 1.019 | 1.018-1.019 | <0.001 |
| BUN (incremented by 1 mmol/L) | 1.174 | 1.168-1.179 | <0.001 |
| Serum uric acid（incremented by 1μmol/L） | 1.004 | 1.004-1.004 | <0.001 |
| LDL-C (incremented by 1 mmol/L) | 1.311 | 1.269-1.354 | <0.001 |
| TG (incremented by 1 mmol/L) | 1.039 | 1.021-1.057 | <0.001 |
| Serum fibrinogen (incremented by 1 g/L) | 1.529 | 1.490-1.569 | <0.001 |
| Urinary protein (ref. 0~±) |  |  |  |
| 1+ – 2+ | 13.640 | 11.990-15.520 | <0.001 |
| 3+ – 4+ | 52.642 | 45.910-60.360 | <0.001 |

Abbreviations: HR: hazard ratio; CI: confidence interval; DR: diabetic retinopathy; GLP1-RA: glucagon-like peptide receptor agonists; RAAS: renin-angiotensin- aldosterone system; HGB: hemoglobin; PLR: platelet/lymphocyte ratio; ALB: albumin; Scr: serum creatinine; BUN: blood urea nitrogen; LDL-C: low-density lipoprotein cholesterol; TG: triglyceride.

**Table S3.** Predictors identified by multivariate Cox regression analysis

| **Predictors** | **HR** | **95%CI** | ***P*-value** |
| --- | --- | --- | --- |
| Age (incremented by 1 year) | 0.993 | 0.988-0.998 | 0.003 |
| Hypertension | 1.651 | 1.446-1.885 | <0.001 |
| DR | 1.433 | 1.280-1.604 | <0.001 |
| HGB (incremented by 1 g/L) | 0.982 | 0.980-0.985 | <0.001 |
| Serum ALB (incremented by 1 g/L) | 0.962 | 0.954-0.971 | <0.001 |
| Scr (incremented by 1 μmol/L) | 1.009 | 1.008-1.010 | <0.001 |
| Serum uric acid (incremented by 1 μmol/L) | 1.001 | 1.000-1.002 | <0.001 |
| LDL-C (incremented by 1 mmol/L) | 1.091 | 1.051-1.133 | <0.001 |
| Serum fibrinogen (incremented by 1 g/L) | 1.055 | 1.018-1.093 | 0.004 |
| Urinary protein 1+ – 2+ | 4.608 | 3.981-5.333 | <0.001 |
| Urinary protein 3+ – 4+ | 7.647 | 6.395-9.144 | <0.001 |

Abbreviations: HR: hazard ratio; CI: confidence interval; DR: diabetic retinopathy; HGB: hemoglobin; ALB, albumin; Scr, serum creatinine; LDL-C: low density lipoprotein cholesterol.

**Table S4.** Baseline clinical characteristics of the study population in the external validation cohort

| **Characteristics** | **Overall**  **(n=25,745)** | **Patients**  **without outcome**  **(n=24,661)** | **Patients**  **with outcome**  **(n=1,084)** | ***P*-value** |
| --- | --- | --- | --- | --- |
| **Demographics** | | | | |
| Age (years) | 63 ± 11 | 63 ± 11 | 61 ± 11 | <0.001 |
| Sex (male %) | 55.37 | 57.89 | 63.75 | <0.001 |
| **Complications or Comorbidities** | | | | |
| DR (%) | 10.10 | 9.36 | 26.85 | <0.001 |
| Diabetic peripheral neuropathy (%) | 11.83 | 11.14 | 27.49 | <0.001 |
| Diabetic vascular disease (%) | 9.85 | 9.33 | 21.86 | <0.001 |
| Hypertension (%) | 55.37 | 54.47 | 75.74 | <0.001 |
| CHD (%) | 21.02 | 21.10 | 19.28 | 0.162 |
| Cerebral vascular disease (%) | 17.98 | 18.11 | 15.04 | 0.011 |
| **Treatment method** | | | | |
| Insulin (%) | 29.23 | 28.60 | 43.36 | <0.001 |
| DPP-4 inhibitors (%) | 1.17 | 1.13 | 2.21 | 0.002 |
| GLP1-RA (%) | 0.10 | 0.10 | 0.28 | 0.191 |
| SGLT-2 inhibitors (%) | 0.03 | 0.04 | 0.00 | 1.000 |
| RAAS inhibitors (%) | 15.37 | 14.80 | 28.32 | <0.001 |
| Lipid-lowering agents (%) | 18.87 | 18.45 | 28.32 | <0.001 |
| **Clinical characteristics** | | | | |
| SBP (mmHg) | 133 (120, 148) | 132 (120, 147) | 145 (130, 163) | <0.001 |
| DBP (mmHg) | 78 (70, 86) | 78 (70, 86) | 80 (72, 90) | <0.001 |
| HGB (g/L) | 127.02 ± 22.50 | 127.90 ± 22.05 | 106.94 ± 23.40 | <0.001 |
| Neutrophils (×10^9^/L) | 5.21 ± 3.62 | 5.18 ± 3.62 | 5.80 ± 3.67 | <0.001 |
| Lymphocytes (×10^9^/L) | 1.69 ± 1.46 | 1.70 ± 1.48 | 1.52 ± 0.92 | <0.001 |
| Platelets (×10^9^/L) | 201.05 ± 84.87 | 200.34 ± 84.25 | 217.30 ± 96.43 | <0.001 |
| NLR | 2.71 (1.84, 4.43) | 3.00 (2.00, 4.00) | 3.44 (2.40, 5.71) | <0.001 |
| PLR | 118.42  (87.02, 169.00) | 118.00  (87.00, 167.00) | 146.04  (105.24, 210.00) | <0.001 |
| Serum fibrinogen (g/L) | 3.61 ± 1.34 | 3.57 ± 1.33 | 4.43 ± 1.42 | <0.001 |
| Serum ALB (g/L) | 38.87 ± 5.91 | 39.11 ± 5.75 | 33.26 ± 6.74 | <0.001 |
| Scr (μmol/L) | 79.91 ± 41.50 | 76.22 ± 34.25 | 163.74 ± 82.92 | <0.001 |
| BUN (mmol/L) | 6.17 ± 3.19 | 5.96 ± 2.84 | 11.05 ± 5.72 | <0.001 |
| Serum uric acid (μmol/L) | 337.48 ± 112.94 | 334.56 ± 111.43 | 403.87 ± 126.03 | <0.001 |
| Serum CysC (g/L) | 1.07 ± 0.56 | 1.03 ± 0.47 | 2.11 ± 1.10 | <0.001 |
| eGFR (mL/min/1.73 m^2^) | 87.34±26.58 | 89.03±25.01 | 49.09±32.09 | <0.001 |
| LDL-C (mmol/L) | 2.61 ± 1.04 | 2.60 ± 1.03 | 2.83 ± 1.26 | <0.001 |
| HDL-C (mmol/L) | 1.06 ± 0.35 | 1.06 ± 0.35 | 1.07 ± 0.43 | 0.795 |
| TC (mmol/L) | 4.49 ± 1.52 | 4.47 ± 1.50 | 4.86 ± 1.91 | <0.001 |
| TG (mmol/L) | 1.44 (1.03, 1.94) | 1.44 (1.03, 2.12) | 1.64 (1.11, 2.41) | 0.003 |
| FBG (mmol/L) | 9.77 ± 5.20 | 9.77 ± 5.17 | 9.79 ± 5.88 | 0.904 |
| HbA1c (%) | 7.94 ± 2.13 | 7.94 ± 2.12 | 8.11 ± 2.50 | 0.028 |
| Urinary protein |  |  |  |  |
| 0 or ± (%) | 80.62 | 82.70 | 33.30 | <0.001 |
| 1+ or 2+ (%) | 15.70 | 14.71 | 38.19 | <0.001 |
| 3+ or 4+ (%) | 3.68 | 2.59 | 28.51 | <0.001 |

Abbreviations: DR: diabetic retinopathy; CHD: coronary heart disease; DPP-4: dipeptidyl peptidase-4; GLP1-RA: glucagon-like peptide receptor agonists; SGLT-2: sodium-glucose co-transporter 2; RAAS: renin-angiotensin-aldosterone system; SBP: systolic blood pressure; DBP: diastolic blood pressure; HGB: hemoglobin; NLR: neutrophil/lymphocyte ratio; PLR: platelet/lymphocyte ratio; ALB: albumin; Scr: serum creatinine; BUN: blood urea nitrogen; CysC: cystatin C; eGFR: estimated glomerular filtration rate; LDL-C: low-density lipoprotein cholesterol; HDL-C: high-density lipoprotein cholesterol; TC: total cholesterol; TG: triglyceride; FBG: fasting blood glucose; HbA1c: glycated hemoglobin.

**Table S5.** The optimal cut-off value of each continuous variable obtained by the minimum *P*-value approach

| **Continuous variables** | **The optimal cut-off value** |
| --- | --- |
| Age (year) | 56 |
| HGB (g/L) | 108 |
| Serum ALB (g/L) | 33 |
| Scr (μmol/L) | 115 |
| Serum uric acid (μmol/L) | 435 |
| LDL-C (mmol/L) | 4 |
| Serum fibrinogen (g/L) | 4 |

Abbreviations: HGB: hemoglobin; ALB: albumin; Scr: serum creatinine; LDL-C: low-density lipoprotein cholesterol.

**Table S6.** The HR value and corresponding score of each variable in the risk score

| **Predictors** | **HR (95%CI)** | ***P*-value** | **Point** |
| --- | --- | --- | --- |
| Age (year) |  |  |  |
| ≥56 (ref) |  |  |  |
| <56 | 1.20 (1.07-1.34) | 0.002 | 1 |
| Hypertension |  |  |  |
| No (ref) |  |  |  |
| Yes | 1.62 (1.42-1.85) | <0.001 | 2 |
| DR |  |  |  |
| No (ref) |  |  |  |
| Yes | 1.38 (1.23-1.55) | <0.001 | 1 |
| HGB (g/L) |  |  |  |
| ≥108 (ref) |  |  |  |
| <108 | 2.11 (1.89-2.36) | <0.001 | 2 |
| Serum ALB (g/L) |  |  |  |
| ≥33 (ref) |  |  |  |
| <33 | 1.75 (1.56-1.96) | <0.001 | 2 |
| Scr (μmol/L) |  |  |  |
| <115 (ref) |  |  |  |
| ≥115 | 5.12 (4.48-5.87) | <0.001 | 5 |
| Serum uric acid (μmol/L) |  |  |  |
| <435 (ref) |  |  |  |
| ≥435 | 1.32 (1.19-1.47) | <0.001 | 1 |
| LDL-C (mmol/L) |  |  |  |
| <3.4 (ref) |  |  |  |
| ≥3.4 | 1.27 (1.14-1.42) | <0.001 | 1 |
| Serum fibrinogen (g/L) |  |  |  |
| <4 (ref) |  |  |  |
| ≥4 | 1.27 (1.13-1.42) | <0.001 | 1 |
| Urinary protein |  |  |  |
| 0 or ± (ref) |  |  |  |
| 1+ or 2+ | 4.31 (3.73-4.99) | <0.001 | 4 |
| 3+ or 4+ | 8.21 (6.92-9.75) | <0.001 | 8 |

Abbreviations: DR: diabetic retinopathy; HGB: hemoglobin; ALB: albumin; Scr: serum creatinine; LDL-C: low-density lipoprotein cholesterol.
